# Coffee-Derived Exosome-Like Nanoparticles: Are They the Secret Heroes?

**DOI:** 10.5152/tjg.2022.21895

**Published:** 2023-02-01

**Authors:** Murat Kantarcıoğlu, Gülşen Yıldırım, Pınar Akpınar Oktar, Serpil Yanbakan, Zeynep Büşra Özer, Deniz Yurtsever Sarıca, Serpil Taşdelen, Emel Bayrak, Dilara Fatma Akın Balı, Seçkin Öztürk, Kamil Can Akçalı, Üstün Ezer, Ahmet Emin Kürekçi

**Affiliations:** 1 LÖSEV LÖSANTE Hospital, Ankara, Turkey; 2 Ankara University Stem Cell Institute, Ankara, Turkey; 3 Department of Medical Biology, Niğde Ömer Halis University Faculty of Medicine, Niğde, Turkey; 4 Middle East Technical University, Ankara, Turkey

**Keywords:** Coffee edible plant derived exosome-like nanopar­ticles, coffee drink, exosomes, hepatocellular carcinoma, size exclusion chromatography

## Abstract

**Background:**

Regular coffee consumption has beneficial and preventative effects on liver and chronic neurodegenerative diseases. However, the studies performed with the ingredients found in coffee beverages have not clarified the responsible mechanisms. Exosomes are small, membrane-coated cargo packages secreted by prokaryote and eukaryote cells. Exosomes regulate intercellular communication and affect cellular metabolic activities even among different species. In this study, we aimed to isolate and characterize the edible plant-derived exosome-like nanoparticles from roasted hot coffee beverages, hypothesizing that the edible plant-derived exosome-like nanoparticles were responsible for the beneficial effects of coffee.

**Methods:**

Size exclusion chromatography and commercial kits were used for the isolation process. Efficient coffee edible plant-derived exosome-like nanoparticle fractions were determined by an ultraviolet-visible spectrophotometer. Harvested coffee edible plant-derived exosome-like nanoparticles were characterized by transmission electron microscopy. The quantification procedure was performed using a commercial kit. Coffee edible plant-derived exosome-like nanoparticles’ proliferative effects on human hepatic stellate cells and human hepatocellular carcinoma cells were studied using an MTT (3-(4,5-Dimethylthiazol-2-yl)-2,5-Diphenyltetrazolium Bromide) assay. Whole-exosome RNA sequencing was performed.

**Results:**

Transmission electron microscopy scanning analysis indicated round-shaped nanoparticles with sizes ranging from 40 to 100 nm. Both size exclusion chromatography and kit-isolated edible plant-derived exosome-like nanoparticle samples showed maximum absorbance at 227.5 nm in ultraviolet-visible spectrophotometer analysis. Regarding the quantitation results, kit isolation was more efficient than the size exclusion chromatography method when the harvested particle numbers were compared. An important MTT assay finding confirmed the observed beneficial effects of coffee beverages: coffee edible plant-derived exosome-like nanoparticles significantly suppressed hepatocellular carcinoma cell proliferation. As a result of sequencing, we identified 15 mature miRNAs. A MapReduce-based MicroRNA Target Prediction Method (The DIANA tools’ MR-microT algorithm) highlighted 2 genes specifically associated with the miRNAs that we obtained: KMT2C and ZNF773.

**Conclusion:**

For the first time in the literature, coffee edible plant-derived exosome-like nanoparticles were identified. These nanoparticles may have therapeutic effects on chronic liver diseases. Experimental studies, therefore, should be performed on disease models to demonstrate their efficacy.

**Main Points**

- For the first time in literature, coffee edible plant-derived exosome-like nanoparticles (EPDENs) were discovered.
- Whole-exosome sequencing determined 15 novel miRNAs.
- Coffee EPDENs suppress hepatocellular carcinoma proliferation.
- Coffee EPDENs seem to be responsible for the beneficial effects of coffee.

## Introduction

Coffee, the most consumed hot beverage globally, has recently gained increased prominence due to its proven health benefits. Coffee consumption is inversely associated with total and cause-specific mortality,^1^ and the effects of coffee consumption on the liver are noteworthy. Meta-analyses have revealed the beneficial and protective effects of coffee on hepatic fibrosis and cirrhosis in patients with chronic liver disease^2^ and even on long-term survival following liver transplantation.^3^ Another organ in which the positive effects of coffee have been observed is the brain. Alzheimer’s disease is among the most common neurodegenerative disorders, and its prevalence in the world’s aging population has increased.^4^ The results of meta-analyses have provided motivation for the regular consumption of coffee to help reduce the likelihood of Alzheimer’s disease, as well as to help prevent other neurodegenerative disorders like dementia and Parkinson’s disease.^5^ Nevertheless, studies performed using ingredients such as caffeine, caffeoyl, kahweol, and trigonelline have not been able to elucidate the mechanisms responsible for these beneficial effects.^6^

Exosomes are small vesicles with a diameter ranging from 40 to 100 nm. They are formed within endosomal compartments and secreted through the fusion of multivesicular bodies with the plasma membrane.^7,8^ These tiny membrane-coated cargo packages are secreted by prokaryotic and eukaryotic cells and transport proteins, lipids, and nucleic acids to other cells.^9^ Exosomes regulate intercellular communication and affect cellular metabolic activities, even between different species. In an experimental model, mouse mast cell-derived exosomes transported to human mast cells induced the expression of mouse proteins in the donor cells.^10^ Exosome release in plant cells was demonstrated more than a decade before it was isolated from mammalian cells;^11^ however, researchers only started isolating and characterizing edible plant-derived exosome-like nanoparticles (EPDENs) from ginger, carrots, grapefruit, and grapes in 2014.^12^ Their data revealed the important role that EPDENs play in terms of maintaining intestinal homeostasis.

Most chronic liver diseases stemming from different etiologies result in progressive liver fibrosis. Myofibroblasts produce an extracellular matrix, which includes type I collagen and is responsible for the development of fibrous scarring in liver fibrosis.^13^ A normal liver has a small amount of type I collagen and no detectable myofibroblasts; however, myofibroblasts can appear early when the liver is injured. The specific marker for myofibroblasts is alpha-smooth muscle actin (α-SMA) protein, which is the actin isoform that predominates within vascular smooth muscle cells and plays an important part in fibrogenesis.^14^ The authors of a comprehensive review reported associations between coffee consumption and changes in liver enzymes, liver fibrosis, and hepatocellular carcinoma (HCC) in patients with a variety of chronic liver diseases.^15^ They pointed out that coffee consumption is inversely related to each of these outcomes, and a dose–response relationship exists. We therefore aimed to isolate and characterize the coffee EPDENs from a hot roasted coffee drink, which we hypothesized as responsible for the beneficial effects of coffee. Accordingly, we evaluated the effects of coffee EPDENs on human HCC cell proliferation and on the expression of α-SMA transcripts, human hepatic stellate cell line.

## Materials and Methods

### Isolation of Coffee EPDENs

All the reagents used were analytical-reagent grade. ELGA-Q water (18.2 MΩ cm^−1^; ELGA Purelab OptionQ, Woodridge, UK) was used where necessary in all the experiments.

A 35 g roasted ground Arabica coffee sample (Tchibo Privat Kaffee, Latin Grande, Guatemala) was brewed in 200 mL deionized hot water (65°C) and stirred for 8 minutes. The infused coffee sample was filtered consecutively through coarse filter paper and 0.45 µm pore size membrane filter paper (Isolab, Eschau, Germany)

Two different coffee EPDEN isolation methods, namely, size exclusion chromatography (SEC) and a commercial kit, were used. The SEC followed the method described by Böing et al.^16,17^ An SEC column with a diameter of 1 cm was stacked with 15 mL Sepharose^®^ CL-6B (Sigma–Aldrich, Merck KGaA, Darmstadt, Germany). Subsequently, the Sepharose CL-6B was washed with 0.01 M phosphate buffered saline (PBS; Sigma–Aldrich). Then, 1 mL of coffee was loaded on the column, followed by elution with 25 mL of PBS (0.01 M). The eluent was collected in 25 fractions from the column at 1 mL per Eppendorf tube (Merck KGaA). The fractions between 7 and 18 of the coffee samples were collected in a falcon tube (Isolab, Eschau, Germany) as described previously. The glass chromatography columns (300 × 10 mm) were purchased from Isolab (Eschau, Germany). The Sartorius model ED224S (Goettingen, Germany) analytical balance and Labnet S0200 Vortex Mixer (NJ) were used.

In the second EPDEN isolation process, Urine Exosome Purification Midi Kit (Cat. 57800) was used as described in the kit protocol manual and was purchased from Norgen Biotek Corp (Thorold, Ontario, Canada). The ethanol (96%) used during the extractions was purchased from Merck (Darmstadt, Germany).

The isolated EPDENs were stored at +4°C for further experiments, including ultraviolet-visible (UV/Vis) spectrophotometer analysis, transmission electron microscopy (TEM) analysis, quantitation, and microRNA (miRNA) analysis.

### Exploration of Coffee EPDEN Fractions Using UV/Vis Spectrophotometry

To determine the most efficient range of isolated EPDEN fractions during column elution, an Agilent Cary 60 UV-Vis spectrophotometer (Santa Clara, Calif, USA) and Jel electrophoresis (Biometra, Compact XS, Endress+Hauser AG, Switzerland) were used.

### Characterization of Coffee EPDENs by Transmission Electron Microscopy

Transmission electron microscopy is frequently used to characterize exosomes. This method enables the detection and characterization of particles down to an imaging resolution of ~1 nm.^18^ The coffee EPDEN samples isolated using both the SEC and kit methods were stained with 2% (w/v) uranyl acetate and fixation. There, the samples were mounted onto grids. Each grid was then studied with TEM and imaged to determine the size and morphology. The images were acquired using an FEI/Tecnai G2 Spirit model BioTwin TEM operating at 120 kV and mounted with an FEI Eagle camera (FEI Company, Waltham, Mass, USA).

### Coffee EPDEN Quantitation

An EXOCET exosome quantitation kit (Palo Alto, Santa Clara, Calif, USA) was used in accordance with the manufacturer’s recommended protocols for quantifying exosome-like nanoparticles obtained from roasted filter coffee column and kit isolations. The EXOCET assay, which is enzymatic and colorimetric, was evaluated and read at an optical density (OD) of 405 nm. A standard curve calibrated for isolated EPDENs by NanoSight analysis was included in the kit.

### MTT Cell Proliferation Assay

The MTT assay is a versatile and popular colorimetric cell viability assay.^19^ We investigated the proliferative effects of the coffee EPDENs on a human hepatic stellate cell line (LX-2) and HCC cell line (Hep 40 cells). Both the LX-2 and Hep 40 cells were purchased from Sigma–Aldrich. First, 1 × 10^4^ cells/well were seeded into a 96-well plate. The cells were then incubated at 37°C for 24-72 hours to determine the optimal incubation time. An MTT Cell Growth Assay Kit (Merck, Penzberg, Germany) was used in accordance with the manufacturer’s instructions. The OD values at 570 nm were read using an Epoch microplate reader (Biotek, Winooski, Vt, USA).

To determine the optimal dose (particles/microliter), we analyzed 10 different concentrations of coffee EPDENs on the LX-2 cells (8 × 10^7^, 16 × 10^7^, 24 × 10^7^, 32 × 10^7^, 4 × 10^8^, 48 × 10^7^, 56 × 10^7^, 64 × 10^7^, 72 × 10^7^, and 8 × 10^8^ particles/µL). The incubation periods range from 24 to 72 hours. Using the MTT assay results we obtained from the LX-2 cell series, we determined 4 different concentrations of the HCC cells, namely, 2 × 10^8^, 4 × 10^8^, 6 × 10^8^, and 8 × 10^8^ particles/µL.

### RNA Isolation and α-SMA Expression—Quantitative Real-Time Polymerase Chain Reaction

The total RNA isolation from the human LX-2 cells and Hep 40 cells at the end of the 72-hour treatment was performed using the RiboEx Total RNA Solution (GeneAll, Lisbon, Portugal) in accordance with the manufacturer’s instructions. cDNA synthesis was performed using a Fast SCRIPT cDNA synthesis kit (TONBO Biosciences, San Diego, Calif, USA). The cDNA was amplified with the GoTaq qPCR master mix (Promega, Madison, Wis, USA). The expression of the α-SMA mRNA was normalized according to the glyceraldehyde 3-phosphate dehydrogenase (GAPDH) expression level. Fold changes in the expression of the genes were analyzed using the comparative (2^−ΔΔCt^) method. An untreated control group was used as the calibrator. The primer sequences used in this study were GAPDH (forward 5’-GGCTGAGAACGGGAAGCTTGTCAT-3’; reverse 5’-CAGCCTTCTCCATGGTGGTGAAGA-3’) and α-SMA (forward 5’-TATCAGGGGGCACCACTATG-3’; reverse 5’-GCTGGAAGGTGGACAGAGAG-3’).

The statistical significances between the quantitative real-time polymerase chain reaction (qRT-PCR) groups were determined using a paired Student’s *t*-test. The values from the qRT-PCR were expressed as means ± standard deviation (SD) (n = 3). *P* < .05 was considered statistically significant.

### Statistical Analysis

The statistical significance between the groups of qRT-PCR and MTT assay was determined by the unpaired Student’s *t*-test. The values from qRT-PCR and MTT were expressed as the means ± SD (n = 3). “N” indicated the number of biological replicates. *P* < .05 was chosen statistically significant. All calculations were performed with GraphPad Prism 8 software (San Diego, Calif, USA).

### Determination of the EPDENs RNA Sequence and Identification of Possible miRNAs

The whole-exosome RNA sequencing was performed by an outsourced commercial firm. We used the exosome RNA Purification Midi Kit (Nogen, Thorold, Canada) to isolate the RNA from the coffee EPDENs harvested, using the SEC column method. MicroRNAs are small non-coding RNAs consisting of 18-22 nucleotides that play a particularly important role in the regulation of gene expression at a post-transcriptional level.^20^ The SRNA-seq data were obtained by RNA sequencing. The miRNAs were identified directly using the BrumiR tool, which is a de novo algorithm based on the Bruijn approach.^21^

## Results and Discussion

The ingredients responsible for coffee’s benefits and the cellular pathways they affect are unclear. Coffee has many components, including hydroxycinnamic acids, flavonoids, tocopherols, diterpene alcohols, melanoidins, and chlorogenic acids. Among the ingredients in coffee, caffeine has received particular interest and attention.^22^ The protective effects of coffee, especially in chronic neurodegenerative diseases, have been attributed to the caffeine it contains. However, recent evidence has suggested that decaffeinated coffee can also be highly effective.^23^

We used an alternative approach to reveal the cause of the beneficial effects of coffee. Studies on the exosome-like nanoparticles of some edible plant products and their metabolic molecular effects have been conducted.^12^ However, to the best of our knowledge, no reports on coffee-derived exosome-like nanoparticles have been published in the literature to date. For the first time, we isolated the extracellular vesicles compatible with exosome morphology from a hot coffee drink. Among the described protocols for extracellular vesicle isolation, such as ultracentrifugation, filtration, immunoaffinity isolation, polymeric precipitation isolation, and liquid chromatography techniques, we selected SEC.^24^ Exosome-like nanoparticles were isolated from the coffee sample via hydrophobic interaction chromatography using Sepharose CL-6B, and appropriate fractions were decided based on our experimental findings. The eluent was collected in 25 fractions from the column as 1 mL per tube. All the fractions between 7 and 18 of the coffee samples were collected in a falcon tube.

**Figure 1.**
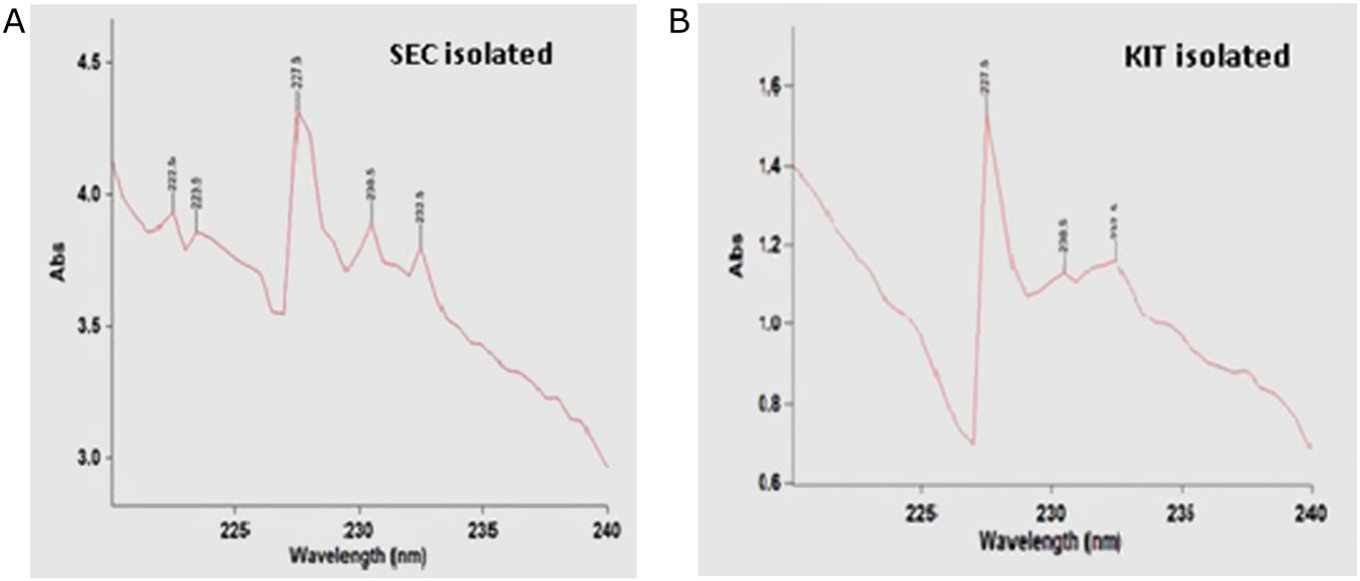
UV-Vis spectrophotometer results for (A) the SEC-isolated and (B) kit-isolated exosome-like nanoparticles of roasted ground coffee.

Using UV-Vis spectrophotometry, the absorption spectra of each fraction collected from the SEC were examined. After comparing the spectra obtained in the EPDEN absorption band in the range 220-240 nm, the fractions between 7 and 18 were chosen for further study. The typical result for a single fraction with column isolation and whole kit isolation of the UV-Vis spectrophotometer is shown in Figure 1.

A coffee bean is exposed to high heat twice during its journey, which starts on the branch of a tree and ends in a cup. The presence of EPDENs in hot coffee is clear evidence that these structures are resistant to heat. This begs the question: what about green coffee? To determine the “roasting heat” sensitive vesicles in green coffee beans, the same study protocol could be performed using a ground green coffee drink to complement our study.

**Figure 2.**
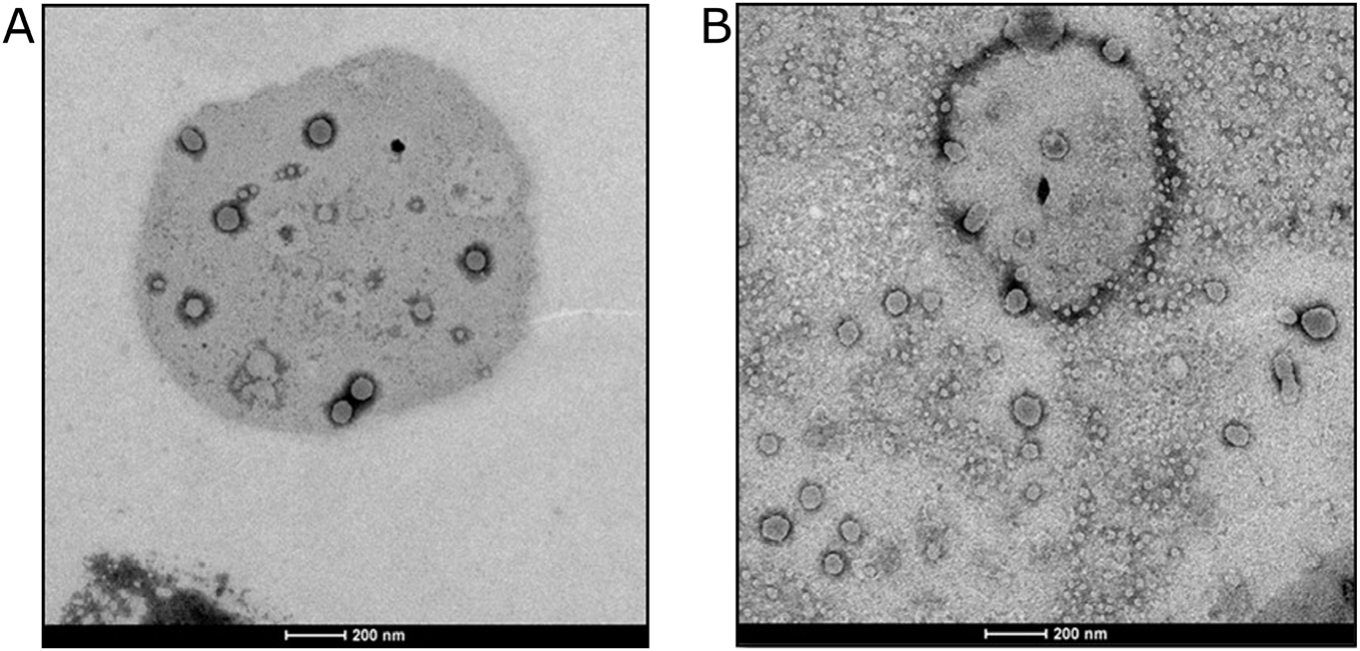
TEM images of (A) the SEC-isolated and (B) kit-isolated exosome-like nanoparticles of roasted ground coffee. TEM, transmission electron microscopy; SEC, size exclusion chromatography.

Xiao et al^25^ described EPDENs that look like exosomes from a structural perspective. Edible plant-derived exosome-like nanoparticles are naturally occurring plant ultra-structures with sizes ranging between 30 and 150 nm.^25^ A TEM imaging study performed in a University Central Laboratory revealed that the nanoparticles we harvested were compatible with the exosomes in terms of the described sizes. The SEC and kit roasted ground coffee-derived vesicle sizes ranged between 10 and 80 nm, as presented in Figures 2a and 2b.

The quantitation kit OD (405 nm) results are presented in Table 1. The quantitation process helped determine the minimum number of EPDENs that should be ingested daily. To obtain the benefits of coffee, it is recommended that at least 2 cups be consumed daily, and no upper limit is specified.^23^ The quantification study and TEM images showed that a larger number of particles could be obtained using the kit method.

**Figure 3.**
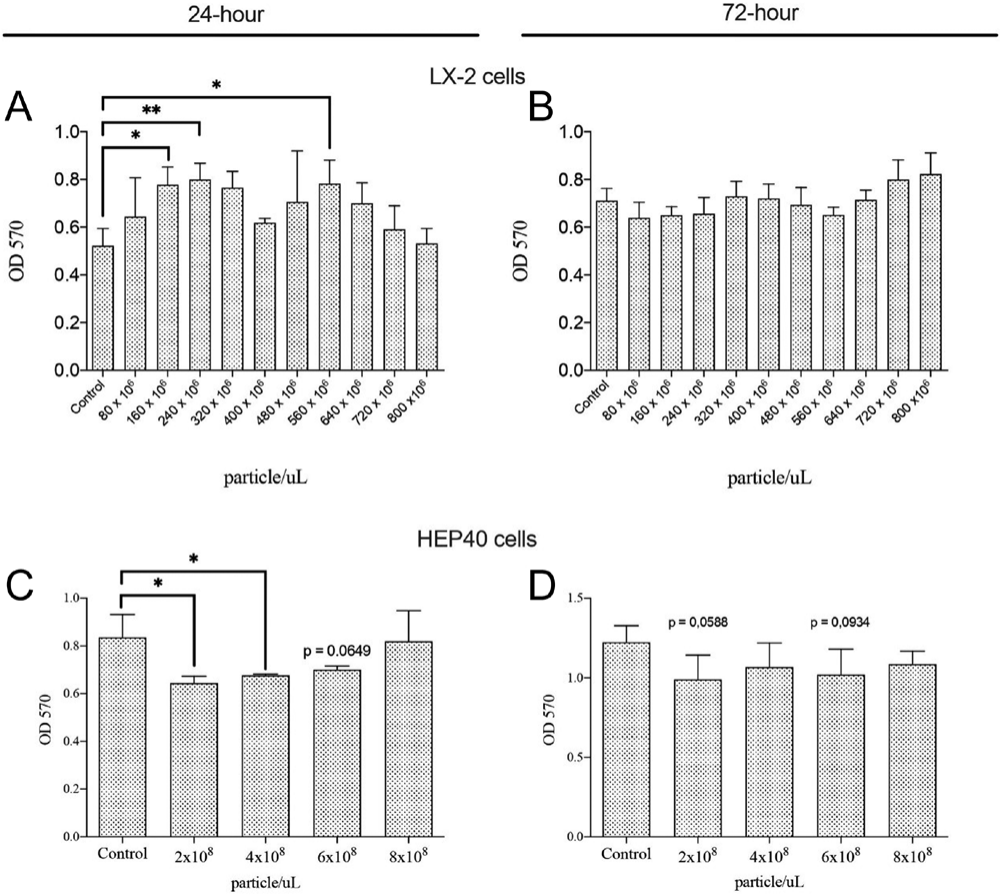
The effects of coffee EPDENs concentrations at 8 × 10^7^, 16 × 10^7^, 24 × 10^7^, 32 × 10^7^, 4 × 10^8^, 48 × 10^7^, 56 × 10^7^, 64 × 10^7^, 72 × 10^7^, and 8 × 10^8^ particles/µL on LX-2 cells (A–B) and 2 × 10^8^, 4 × 10^8^, 6 × 10^8^, and 8 × 10^8^ particles/µL on Hep 40 cells after 24- and 72-hour treatments (C–D). The data represent the mean and SD of the triplicate samples. All the data are represented as the mean ± SD (n = 3). Significance level, ^*^ *P* < .05 versus control group. EPDENs, edible plant-derived exosome-like nanoparticles; SD, standard deviation.

In our MTT study, we used 2 different cell lines; fibrotic (LX2) and hepatocellular (HEP40) cell lines. We chose 24 and 72 hours as early and late response of these cell lines upon EPDENs exposure. As an early response to EPDENs exposure in LX2, we found that between the concentration of 80 and 240 × 10^6^, the proliferation of the cells increased significantly compared to that of control group (Figure 3a). On the other hand, at higher concentrations except 560 × 10^6^, we did not see any significant changes compared to control group. On the other hand, we did not observe any significant effect of EPDENs in the late response in LX2 cells (Figure 3b). Our MTT results for the effect of EPDENs in HEP40 showed a concentration-dependent significant decrease (2-4 × 10^8^) upon exposure to EPDENs in HEP40 cells compared to that of control group in early response (Figure 3c) but not in late response (Figure 3d). This finding was consistent with the reported inverse proportion between caffeinated and decaffeinated coffee consumption and HCC prevalence.^26^ A meta-analysis revealed the ineffectiveness of caffeine in this process. Like that of LX2, the early response has not changed in further increased concentrations. The lack of a significant increase in higher concentrations of EPDENs at early response may be related to the content of these EPDENs. To support this hypothesis, we have identified 15 novel miRNA sequences in the content of the EPDENs (Table 2). Since there is no known in vitro function associated with these miRNAs, we applied an artificial intelligence program; a MapReduce-based microRNA target prediction algorithm for these novel miRNAs. This in silico approach revealed 2 important genes, ZNF773 and KMT2C and their network in liver fibrosis leading to chronic liver disease (Figure 5). It is tempting to speculate that these contents can direct and control the response of EPDENs through the progression of liver fibrosis. It is important to note that this explanation is only applicable for the early response (24 hours) not to late response (72 hours). This is particularly important since fibrosis is a progressive condition and early intervention to this progression is critical, the answer may be in EPDENs. This hypothesis clearly warrants new data to prove which we plan to continue with our future studies.

**Figure 4.**
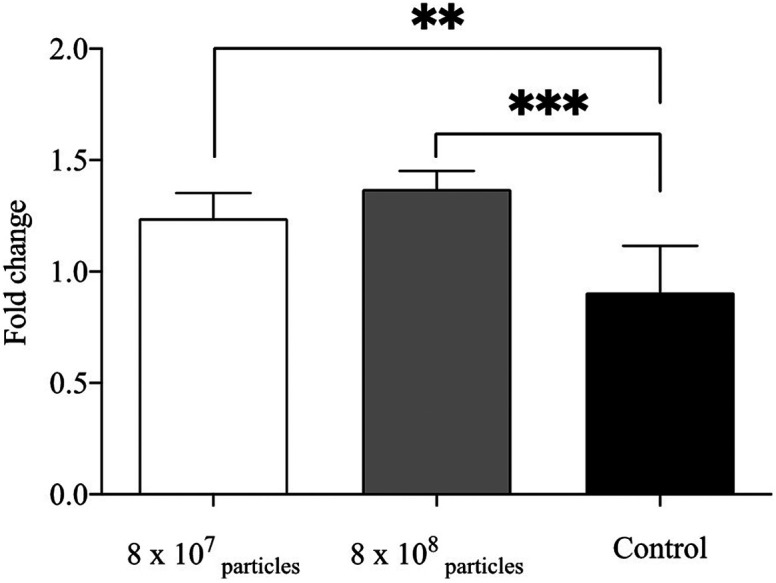
The transcriptional response of α-SMA to coffee EPDEN treatment in LX-2 cells. The error bars reflect the SDs of the results derived from the biological triplicate experiments. The qRT-PCR data are presented as the fold changes of the mRNA levels in the treatment groups relative to the non-treated (control) group based on the normalization against the GAPDH. All the data are presented as mean ± SD. Significance level, ^*^ *P* < .05 versus control group. α-SMA, alpha-smooth muscle actin; EPDENs, edible plant-derived exosome-like nanoparticles; SD, standard deviation; qRT-PCR, quantitative real-time polymerase chain reaction; GAPDH, glyceraldehyde 3-phosphate dehydrogenase.

Since α-SMA is a known marker of liver fibrosis, we checked the mRNA levels of the α-SMA in the LX-2 cells (Figure 4). We used 2 different concentrations (i.e., 8 × 10^7^ and 8 × 10^8^ particles/µL) to further underline how concentrations affect the mRNA expression of α-SMA. Our qRT-PCR results showed that the α-SMA expression levels increased after the administration of the coffee EPDENs in a concentration-dependent manner.

More studies on the use of coffee EPDENs in chronic liver damage and fibrosis that includes animal models therefore need to be performed.

Comprehensive data were obtained after RNA sequencing from the RNAs obtained from the coffee EPDENs. From the obtained sRNA-seq data, miRNAs were identified using the BrumiR tool, a de novo algorithm based on the Bruijn approach.^21^ The mature miRNA sequences isolated from the coffee EPDENs are presented in Table 2.

**Figure 5.**
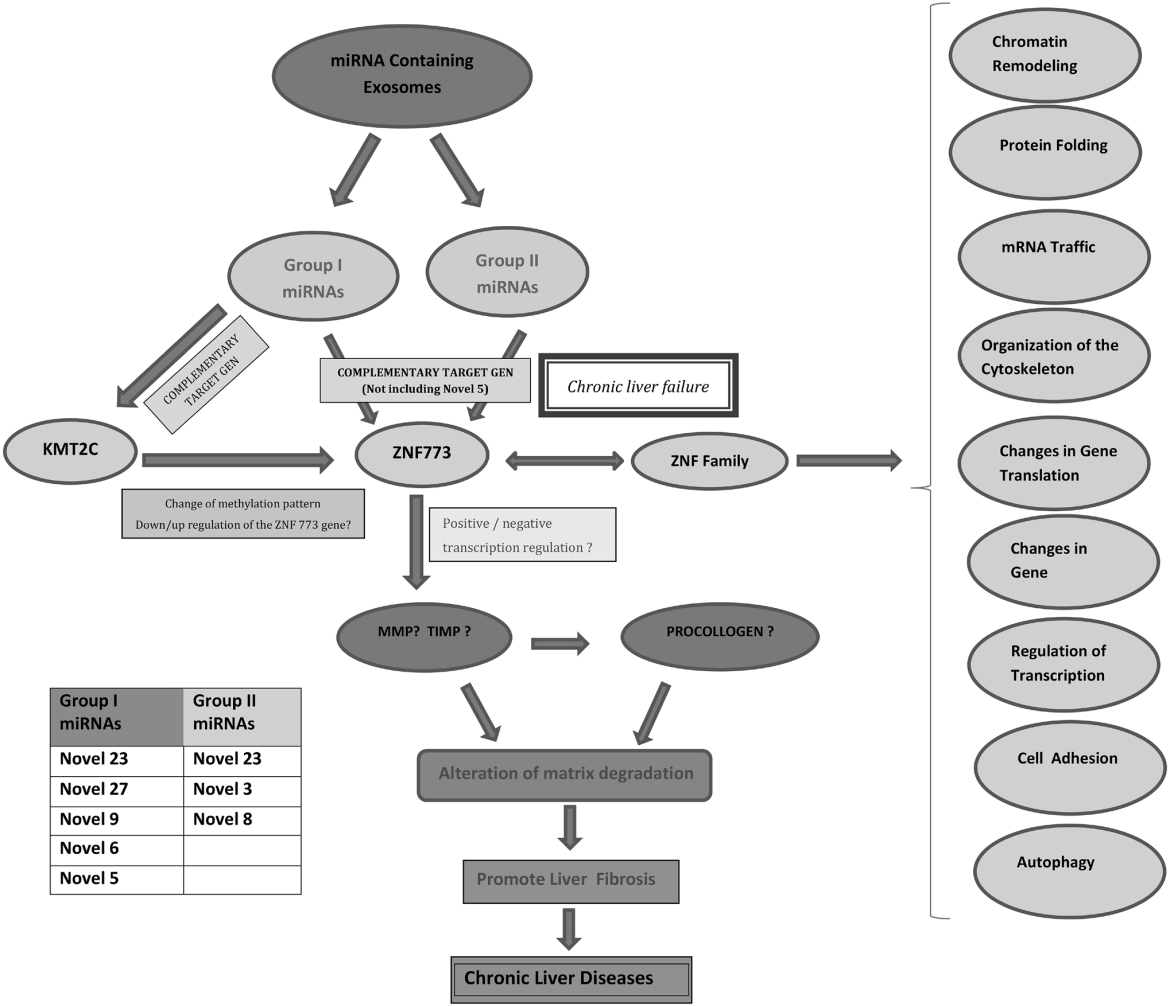
How miRNAs carried by EPDENs in coffee can affect liver fibrosis in chronic liver diseases via ZNF773 and KMT2C genes is schematized using a hypothetical mechanism. KMT2C, lysine methyltransferase 2C; MMP, *matrix metalloproteinase; TIMP, *tissue inhibitor of metalloproteinase; ZNF, zinc finger.

In our study, 2942 target genes were determined from 15 separate miRNAs using the DIANA tool’s MR-microT (a MapReduce-based microRNA target prediction method) algorithm. The score cut-off value was selected using the threshold identifier 0.8.^27,28^ Among these targets, 17 common genes were identified as having coffee EPDEN-derived miRNAs. Each individual coffee EPDEN-derived miRNA was related to a minimum of 4 and a maximum of 7 of these target genes. The DIANA tool’s algorithm highlighted 2 genes (KMT2C, ZNF773), which were specifically associated with the miRNAs we obtained. KMTC2 is a common target gene for 5 miRNAs. KMT2C is a member of the KMT2 (lysine methyltransferase) protein family.^29^ MT2 proteins are histone-modifying actors that play important roles in cell development pathways. Moreover, the repair response to DNA damage is highly managed by KMT2C. With respect to this mechanism, it has been shown that a cell with low KMT2C activity has impaired homologous recombination‐mediated double‐strand break DNA repair abilities. Recent data have emphasized that the downregulation of KMT2C is an accompanying feature in human epithelial cancers.^30^ The other target gene for 7 of the coffee EPDEN-derived miRNAs in our study was ZNF773. There is insufficient information on the functional properties of ZNF773 in the medical literature. According to the hypothetical mechanism we propose, miRNAs ingested with coffee EPDENs may alter the expression of the KMT2C gene, and these changes may affect the ZNF773 gene methylation pattern. In terms of the reported functional features of KMT2C, we therefore schematized a hypothetical mechanism to explain how miRNAs carried by coffee EPDENs can affect liver fibrosis in chronic liver diseases (Figure 5). In 2007, Valadi et al^10^ demonstrated that adding exosomes from mouse mast cells to human mast cell cultures caused these vesicles to fuse with the human cells, and as a result, the human cells began to produce mouse proteins. The same interaction mechanism may be valid for EPDENs. Based on their findings, many researchers have suggested that plant-derived exosome-like particles may enter the cells of different species and affect their metabolic activities.^12^ Plant-derived microRNAs play the main role in this cross-kingdom communication.^31^ A particularly good example of this interaction is MIR168a. Zhang et al^32^ demonstrated the circulation of MIR168a in the sera of Chinese subjects. This exogenous plant miRNA is mostly found in rice. The researchers demonstrated that MIR168a can bind to human/mouse low-density lipoprotein receptor adapter protein 1 mRNA, and eventually LDL removal from mouse plasma decreases. A stunning paper was published by Chuang et al^33^ who found some differentially methylated CpGs located in the genes of people with diseases who benefit from drinking coffee. Coffee consumption was reported to be associated with DNA methylation levels.

We defined 15 novel miRNAs derived from coffee EPDENs that seemed to mostly associate with KMT2C and ZNF773. While KMT2C reportedly provides a protective and repairing response to DNA damage,^30^ not enough information is available about ZNF773. In our bioinformatic study, we have summarized our hypotheses regarding the possible interaction pathways in a diagram (Figure 5).

Due to our limited project budget, we could not study the in vivo effects of coffee EPDEN particles on other cancer cell lines. Genomic and proteomic studies are needed to determine the common target genes of the miRNAs we have obtained. These studies also require funding.

## Conclusion

Coffee EPDENs are a new candidate for plant-derived miRNA-based therapies in chronic liver diseases and HCC. These genetic interactions and functional mechanisms should be explored further in future studies.

## Figures and Tables

**Table 1.** Quantitation Kit Optimal Density (405 nm) Results and Concentrations of Coffee EPDENs in Different Volumes and Diluted in Different Proportions

Coffee EPDEN Sample and Isolation Type	Average OD (405 nm)	Concentration/1 mL	Concentration/200 mL^***^
Filter coffee size exclusion chromatography isolation^*^	0.310	**8 × 10 ^10^**	**16 × 10 ^12^**
Filter coffee kit isolation^**^	0.410	**16 × 10 ^10^**	**32 × 10 ^12^**

^*^The amount of coffee EPDENs obtained by the column sepharose gel method; ^**^The amount of coffee EPDENs obtained with commercial kit (Norgen); ^***^Cup of coffee consumed with daily diet.

OD,optical density.

**Table 2.** Mature MiRNA Sequences Isolated from Coffee EPDENs

MiRNA (Mature_ Identical)	miRNA seq (Mature_ Sequence)
novel_1	ccggugcuggccugcgggc
novel_11	gguaacccgcugaaccuu
novel_14	gaggggaguggcugggga
novel_17	cgagaguuggaccggggg
novel_2	acgcccuugugguuugacu
novel_22	uggacggggucgaugggcgauc
novel_23	aggggagggggcgggcgg
novel_24	aggucacgaguucgagucuc
novel_26	agggugggcaggcuguuaaac
novel_27	cuguggaaccucaugcuu
novel_3	gauggagggacggagagg
novel_5	uuccacagcuuucuugaacuu
novel_6	uggggaggggggcggggc
novel_8	uaugcgugcucacucucuauc
novel_9	ggaggaggaaagagaaagg
